# Staphylococcal superantigen‐like protein 13 activates neutrophils via formyl peptide receptor 2

**DOI:** 10.1111/cmi.12941

**Published:** 2018-09-17

**Authors:** Yuxi Zhao, Kok P. M. van Kessel, Carla J. C. de Haas, Malbert R. C. Rogers, Jos A. G. van Strijp, Pieter‐Jan A. Haas

**Affiliations:** ^1^ Department of Medical Microbiology University Medical Center Utrecht Utrecht The Netherlands

**Keywords:** activation, FPR2, neutrophils, SSL13, *Staphylococcus aureus*

## Abstract

Staphylococcal superantigen‐like (SSL) proteins, one of the major virulence factor families produced by Staphylococcus aureus, were previously demonstrated to be immune evasion molecules that interfere with a variety of innate immune defences. However, in contrast to characterised SSLs, which inhibit immune functions, we show that SSL13 is a strong activator of neutrophils via the formyl peptide receptor 2 (FPR2). Moreover, our data show that SSL13 acts as a chemoattractant and induces degranulation and oxidative burst in neutrophils. As with many other staphylococcal immune evasion proteins, SSL13 shows a high degree of human specificity. SSL13 is not able to efficiently activate mouse neutrophils, hampering in vivo experiments. In conclusion, SSL13 is a neutrophil chemoattractant and activator that acts via FPR2. Therefore, SSL13 is a unique SSL member that does not belong to the immune evasion class but is a pathogen alarming molecule. Our study provides a new concept of SSLs; SSLs not only inhibit host immune processes but also recruit human neutrophils to the site of infection. This new insight allows us to better understand complex interactions between host and *S*. *aureus* pathological processes.

## 1 INTRODUCTION

The Gram‐positive bacterium Staphylococcus aureus (S. aureus) is an opportunistic human pathogen that causes a wide range of diseases from mild skin infections to more serious life‐threatening wound and systemic infections (Spaan, Surewaard, Nijland, & van Strijp, 2013). In order to successfully invade and colonise the human host, S. aureus secretes a large arsenal of immune evasion molecules that specifically target components of the human innate and adaptive immune systems (Foster, 2005; Lewis & Surewaard, 2018; Thammavongsa, Kim, Missiakas, & Schneewind, 2015). These secreted proteins interfere with a range of immune defences, which can be grouped into four categories: blocking, degradation, cell lysis, and modulation (Koymans, Vrieling, Gorham, & van Strijp, 2016). Despite the functional differences and diversity in targets, the staphylococcal immune evasion proteins are secreted proteins that show remarkable resemblances. These proteins contain very conserved structural properties (Jongerius et al., 2007). They are often small, varying in size between 8 and 35 kDa, and have extreme isoelectric points (above 9 or below 5). Another common property of these proteins is that they are located on genomic clusters with other virulence factors. The secretome of S. aureus is predicted to contain up to 270 proteins, of which over 35 staphylococcal evasion molecules have been described (Koymans et al., 2016). Identification and characterisation of these secreted proteins will lead to a better understanding of the S. aureus pathological processes.

Neutrophils play a crucial role in protecting the host from S. aureus infections (van Kessel, Bestebroer, & van Strijp, 2014). Inherited or acquired neutrophil dysfunction, such as leukocyte adhesion deficiency and chronic granulomatous disease, leads to an increased risk of severe S. aureus infections (Voyich et al., 2005). Disruption of physical barriers and invasion of S. aureus initiate the release of proinflammatory signals that promote neutrophil adherence to the vascular endothelium, extravasation, and migration from the bloodstream towards to the site of infection (Spaan, Surewaard, et al., 2013). However, S. aureus can subvert neutrophil functions via the secretion of proteins that inhibit neutrophil recruitment and activation (Bestebroer et al., 2007; Chavakis et al., 2002). A variety of immune evasion proteins have been identified that specifically target neutrophil surface receptors. Some immune evasion proteins inhibit proinflammatory receptors such as chemotaxis inhibitory protein of S. aureus (CHIPS; de Haas et al., 2004), formyl peptide receptor‐like 1 inhibitory protein (FLIPr), and the homologue FLIPr‐like (FLIPrL; Prat et al., 2009; Prat, Bestebroer, de Haas, van Strijp, & van Kessel, 2006). Other immune evasion proteins serve as toxins that use surface receptors to specifically lyse leukocytes, such as the bicomponent toxins (PVL, LukAB, and LukED; Alonzo et al., 2013; DuMont et al., 2013; Spaan, Henry, et al., 2013) and phenol soluble modulins (PSMs; Surewaard et al., 2012). In addition, the PSMs act as potent neutrophil chemoattractants via human formyl peptide receptor 2 (FPR2; Kretschmer et al., 2010; Weiss et al., 2017). The FPR2 is a G protein‐coupled receptor (GPCR) and a member of the FPR family together with the archetype formyl peptide receptor 1 (FPR1). Both receptors are present on neutrophils and myeloid cells and are considered as sensors for microbe‐associated molecular patterns (MAMPs). In contrast to the FPR1, the FPR2 is a promiscuous receptor with various unrelated ligands, which include peptides, parts of proteins, lipids, and small molecules, resulting in different intracellular responses in a ligand‐specific manner (Cattaneo, Parisi, & Ammendola, 2013).

Another group of secreted proteins, of which many are involved in immune evasion, is the staphylococcal superantigen‐like (SSL) proteins (Thammavongsa et al., 2015). SSLs are a family of 14 proteins with structural similarity to staphylococcal superantigens but lack the functional T‐cell receptor binding domain and therefore exhibit no superantigenic activity (Fraser & Proft, 2008). Moreover, structurally, the C‐terminal β‐grasp domain of these SSL proteins show homology to other staphylococcal immune evasion proteins such as CHIPS. SSL1 to SSL11 are encoded on staphylococcal pathogenicity island 2 whereas SSL12, SSL13, and SSL14 are found on the immune evasion cluster 2 (Jongerius et al., 2007; Kuroda et al., 2001). The SSL gene cluster is expressed in all human and animal isolates of S. aureus examined to date, indicating that it is very stable and evolutionary important cluster for the organism (Fitzgerald et al., 2003; Kuroda et al., 2001; Smyth, Meaney, Hartigan, & Smyth, 2007). Furthermore, antibodies against the SSLs are detected in human serum, indicating that they are expressed in vivo and may play a role during infection (Al‐Shangiti, Nair, & Chain, 2005; Fitzgerald et al., 2003). Even though the SSLs are highly conserved and involved in innate immune evasion, they have distinct functions (Fraser & Proft, 2008). It was reported previously that several SSL members located on the main cluster (SSL3, SSL5, SSL6, SSL7, and SSL10) are involved in inhibition of host immune responses (Baker et al., 2007; Bestebroer et al., 2009; Langley et al., 2005; Walenkamp et al., 2009). SSL3 and SSL4 have been described as toll‐like receptor 2 inhibitors and prevent neutrophil activation (Bardoel et al., 2012; Hanzelmann et al., 2016). SSL5 interacts with neutrophil surface receptor CD162 and reduces neutrophil migration (Baker et al., 2007; Bestebroer et al., 2007). SSL6 was identified to interact with CD47 by screening a S. aureus secretome phage display library for binding to isolated human neutrophils (Fevre et al., 2014). SSL7 binds to complement C5 and therefore prevents C5a production (Bestebroer, Aerts, et al., 2010). In addition, SSL7 and SSL10 are associated with blocking complement activation by targeting IgA and IgG, respectively (Bestebroer, Aerts, et al., 2010; Patel, Wines, Langley, & Fraser, 2010). In contrast, none of the SSLs on the minor cluster (SSL12–SSL14) have been functionally characterised.

In this study, we set out to identify new S. aureus proteins that interact with human neutrophils using a S. aureus secretome phage display library. In combination with whole genome sequencing, SSL13 was identified to bind human neutrophils. We show that binding to human neutrophils is FPR2 dependent. Through this interaction, SSL13 activates neutrophils and acts as a chemoattractant. Furthermore, SSL13 induced an oxidative burst and degranulation in neutrophils. In contrast to many other immune evasion proteins that inhibit immune responses, we identified SSL13 as a chemoattractant and a neutrophil activator that acts via the FPR2.

## 2 RESULTS

### 2.1 Phage library sequencing and identification of immune evasion proteins

To identify new S. aureus proteins that interact with human neutrophils using a S. aureus secretome phage display library. The sequencing run produced a total of 1,396 and 23,411 paired‐end reads for the unselected and selection library, respectively. These reads were then quality trimmed using nesoni clip v. 0.128 with the following parameters: –adaptor‐clip yes –match 10 –max‐errors 1 –clip‐ambiguous yes –quality 10 –length 150 (http://www.vicbioinformatics.com/software.nesoni.shtm). About 90% of the read pairs were retained and used for further analyses.

Quality‐trimmed sequence reads were aligned to the Genbank database (accessed on July 20, 2015) using BLAST+ 2.2.31.3 sequences in the nonselected, and four sequences in the selected library did not align with a S. aureus genome and were omitted from analysis. The read frequency was defined as the total count of identical reads. The total amount of unique sequences per annotated gene was defined as number of clones. The highest hit in the unselected library is annotated as a dUTPase with a read frequency of 14 all belonging to a single clone. The 96 reads with the highest read frequency after selection encode for 61 different proteins that are listed in Table S2. There is a large increase in read frequency after selection. The highest read frequency with 883 reads encoding seven unique sequences is annotated as a transmembrane protein involved in mannitol transport. The selection of transmembrane proteins when performing phage display selection on cells was also observed in earlier phage selections in our lab (data not shown). The presence of membranes appear to select for transmembrane domains especially transporter proteins such as ABC transporters. The second highest hit with 196 reads and four different clones identified the recently described S. aureus protein (SPIN) that binds neutrophil MPO and promotes the intracellular survival of S. aureus after phagocytosis (de Jong et al., 2017). Of the total of 61 identified proteins, 12 (20%) were described to play a role in host–microbe interaction. Of these, 11 were already functionally characterised, and for one protein, SSL13, no known function has been described. The fact that SSL13 was identified in this selection suggests that it is involved in binding to neutrophils or its components.

### 2.2 SSL13 specifically interacts with human neutrophils

**Figure 1:**
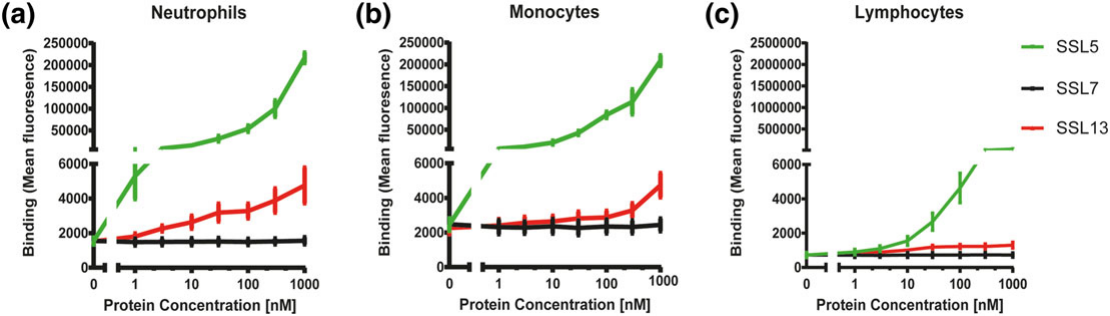
SSL13 binds to human neutrophils, monocytes, but not lymphocytes. Peripheral blood leukocytes were incubated with a threefold dilution series of His‐SSL13 for 30 min at 4°C. Binding was detected with anti‐His‐FITC and analysed by flow cytometry. The different cell populations were identified based on scatter parameters. His‐SSL5 and His‐SSL7 are positive and negative controls for binding, respectively (a–c). Error bars are SEM of three biological replicates analysed in duplicate

To confirm that SSL13 interacts directly with human neutrophils, a threefold dilution series of recombinant SSL13 with an N‐terminal His tag was incubated with human leukocytes isolated from healthy donors. His‐tagged SSL7 and SSL5 were included as negative and positive control neutrophil‐binding proteins, respectively (Bestebroer et al., 2007; Laursen et al., 2010). We observed that SSL13 interacts with human neutrophils and monocytes in a dose‐dependent manner, but no significant binding was observed to lymphocytes (Figure 1a–c).

**Figure 2:**
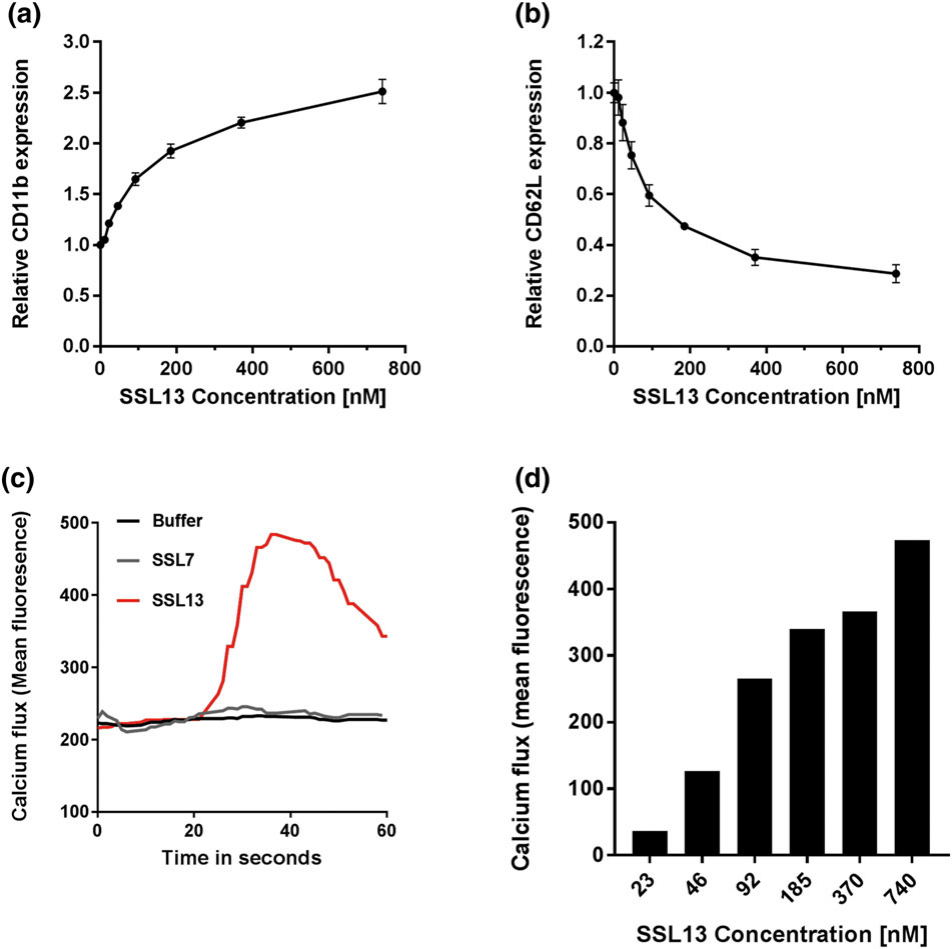
SSL13 activates human neutrophils. Activation of isolated human neutrophils by increasing concentration of SSL13. Increased CD11b expression (a) and decreased CD62L expression (b) are markers for neutrophil activation. Data are mean fluorescence ± SEM of three independent experiments. Addition of SSL13 induces cell activation measured as a transient release of intracellular calcium (c), in a concentration‐dependent manner (d). Data are from one representative experiment

Interestingly, binding experiments conducted at 37°C indicated that SSL13 activates neutrophils as shown by an increase in forward scatter compared with untreated cells (no protein; Cole, Garlick, Galvin, Hawkey, & Robins, 1995; Fletcher & Seligmann, 1985; Figure S2A–C). Activation of neutrophils generally alters the surface expression of major cell adhesion molecules, for example, up‐regulation of CD11b and down‐regulation of CD62L (Jongerius et al., 2007). The effect of SSL13 on CD11b and CD62L expression was evaluated by flow cytometry. We observed that SSL13 enhanced the surface expression of CD11b and simultaneously down‐regulated the expression of CD62L in a dose‐dependent manner (Figure 2a,b). In addition to the altered expression of surface adhesion molecules, activated neutrophils also exhibit intracellular release of calcium (Hamm, 1998). We therefore measured the intracellular release of calcium after neutrophil exposure to a range of SSL13 concentrations (23–740 nM). In concordance with the cell receptor expression assay, our calcium flux data showed that SSL13 induces a transient dose‐dependent release of Ca^2+^ in neutrophils (Figure 2c,d). Degradation of SSL13 by proteinase K completely abolished neutrophil activation indicating that the observed activation is not caused by a nonprotein contaminant in the SSL13 preparation (Figure S3A–D). We used SSL7, which was expressed using the same protein expression system, as a control to rule out any specific activation by contaminant MAMPs such as LPS or formylated peptides. To conclude, SSL13 specifically binds and activates human neutrophils.

### 2.3 SSL13 specifically binds and activates FPR2

**Figure 3:**
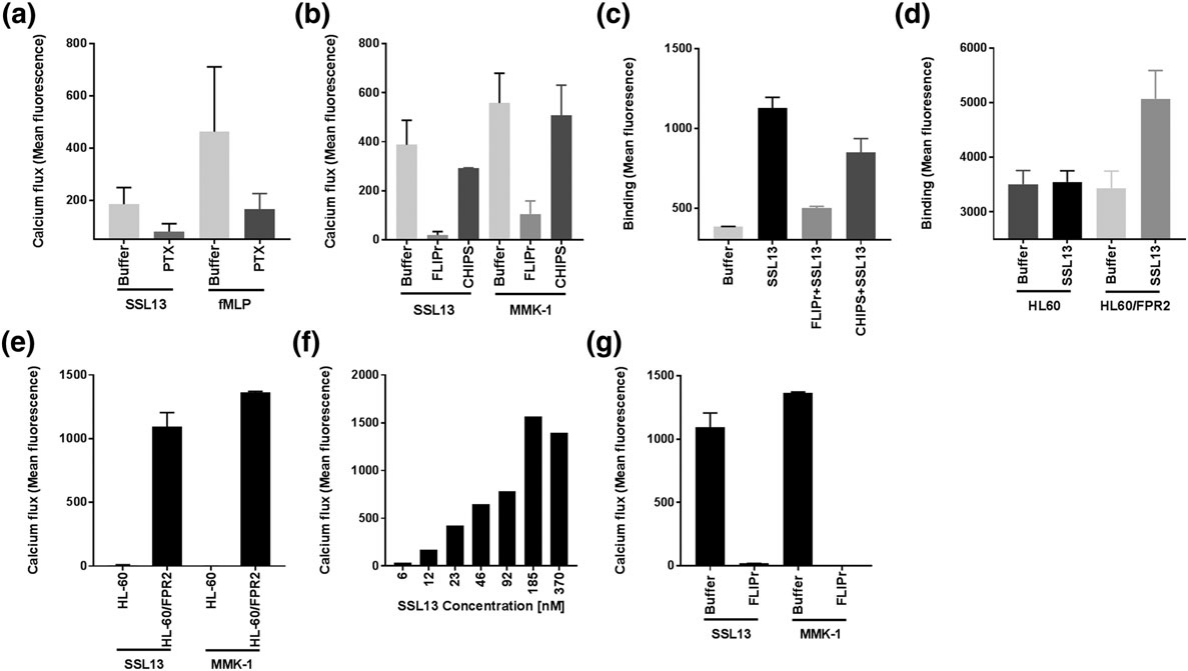
SSL13 specifically binds and activates human formyl peptide receptor 2. (a) Neutrophils were preincubated with or without 3‐μg/ml Pertussis toxin (PTX) for 60 min at 37°C with CO_2_ and then labelled with Fluo‐3‐AM. Neutrophil stimulation by SSL13 is sensitive to PTX. Formyl‐methionyl‐leucyl phenylalanine is the control ligand of formyl peptide receptor 1, which is also sensitive to PTX. (b) Human neutrophil stimulation by SSL13 is inhibited by formyl peptide receptor‐like 1 inhibitory protein (FLIPr), not chemotaxis inhibitory protein of Staphylococcus aureus (CHIPS). (c) SSL13 specific binding to human neutrophils is blocked by FLIPr, not CHIPS. Data represent means ± SEM of three independent experiments. (d) SSL13 specifically binds to formyl peptide receptor 2 (FPR2)‐transfected HL60 cells (HL60/FPR2), but not to control HL60 cells. (e) SSL13 induces profound calcium fluxes in HL60/FPR2 cells, but not in empty HL60 cells. MMK‐1 is a synthetic control ligand of FPR2. (f) HL60/FPR2 cells stimulation by SSL13 is concentration dependent. (g) HL60/FPR2 cells stimulation by SSL13 is sensitive to FPR2‐specific inhibitor FLIPr. Data are mean fluorescence ± SEM of three experiments

As SSL13 induced a rapid and transient release of intracellular Ca^2+^, we examined whether SSL13 acts through a GPCR (Bestebroer, De Haas, & van Strijp, 2010). PTX is a general antagonist of GPCR activation and therefore blocks the release of intracellular Ca^2+^ (Welin et al., 2015). For this purpose, neutrophils were preincubated with or without PTX for 1 hr at 37°C with CO_2_ and then stimulated with 370‐nM SSL13 or fMLP as a reference PTX‐sensitive stimulus (Bokoch & Gilman, 1984; Lad, Olson, & Smiley, 1985). Figure 3a shows that PTX can block both SSL13‐ and fMLP‐induced neutrophil activation, which confirms that SSL13 utilises a PTX‐sensitive GPCR to induce this response.

To further investigate the responsible receptor, a set of well‐characterised agonists and antagonists of neutrophil GPCRs were tested, including those for FPR1 and FPR2, leukotriene B4 receptor (BLTR1), platelet activating factor receptor, complement C5a receptor, and the interleukin 8 receptors CXCR1 and CXCR2. We found that an FPR2 antagonist FLIPr inhibited SSL13‐induced calcium mobilisation, as well as SSL13 binding to human neutrophils (Figure 3b,c). Although FLIPr also slightly inhibited the FPR1 activation, the control protein CHIPS, which specifically inhibits FPR1 (Prat et al., 2009), had no effect on SSL13‐mediated neutrophil activation (Figure 3c). Together, these experiments indicate that SSL13 elicits calcium fluxes in human neutrophils via FPR2.

To further confirm that FPR2 is the receptor for SSL13, we used HL60 cells stably transfected with or without human FPR2 (Christophe, Karlsson, Rabiet, Boulay, & Dahlgren, 2002; Dahlgren et al., 2000). Binding of FITC‐labelled SSL13 was only observed for HL60/FPR2 and not for control HL60 cells (Figure 3d). Furthermore, in order to evaluate the role of FPR2 in recognising SSL13, we analysed the intracellular Ca^2+^ response to SSL13 of HL60 with or without FPR2. Figure 3e shows that SSL13 induces a profound calcium flux in HL60/FPR2 but not in untransfected HL60 cells. The activation potential of SSL13 is comparable with the specific FPR2 agonistic peptide MMK‐1 (Figure 3e). Moreover, SSL13 activated the FPR2‐transfected HL60 cells in a dose‐dependent manner (Figure 3f). Finally, the induced calcium flux of the FPR2‐transfected HL60 cells by SSL13 and MMK‐1 can be inhibited by the FPR2‐specific inhibitor FLIPr (Figure 3g). These findings confirm that SSL13 specifically binds and activates cells via FPR2.

### 2.4 SSL13 is involved in chemoattractant‐induced oxidative burst and degranulation of neutrophils

**Figure 4:**
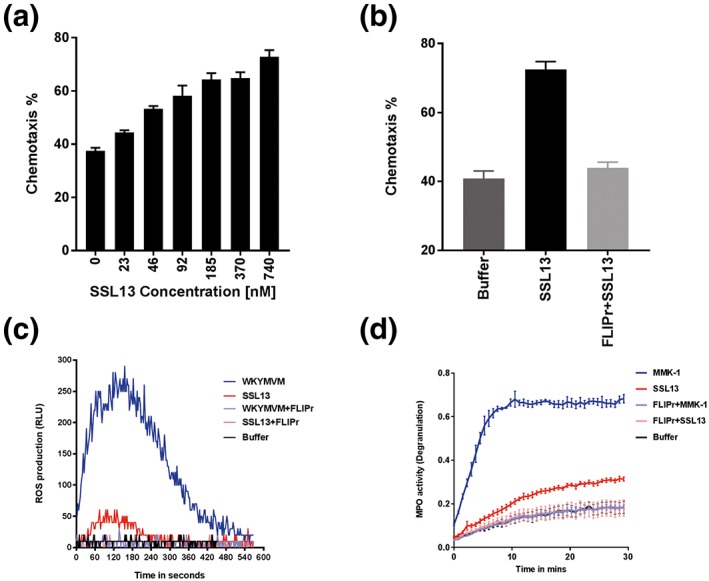
SSL13 is involved in chemoattractant‐induced oxidative burst and degranulation of neutrophils. (a) SSL13 stimulates chemotaxis in human neutrophils in a dose‐dependent manner. (b) SSL13‐induced chemotaxis of human neutrophils is inhibited by the formyl peptide receptor 2 (FPR2) antagonist formyl peptide receptor‐like 1 inhibitory protein (FLIPr). (c) SSL13 stimulates FPR2‐induced oxidative burst. WKYMVM is a synthetic control ligand of FPR2. (d) SSL13 modestly induces neutrophil degranulation via FPR2. MMK‐1 is a positive control. (a,b) Data represent means ± SEM of three experiments. (c,d) Data from a representative experiment. ROS: reactive oxygen species

Triggering FPR2 induces many neutrophil effector functions, including chemotaxis, exocytosis, and superoxide generation (Fu et al., 2006). To investigate whether SSL13 is a chemoattractant, neutrophil migration was measured in a 96‐multiwell transmembrane system. Indeed, SSL13 stimulated chemotaxis of human neutrophils in a dose‐dependent manner (Figure 4a). Moreover, SSL13‐induced chemotaxis in human neutrophils can be blocked by the FPR2 antagonist FLIPr (Figure 4b).

To examine whether SSL13 is involved in FPR2‐induced oxidative burst, a reactive oxygen species (ROS) assay was performed. The peptides WKYMVM and MMK‐1 can both induce FPR2‐mediated ROS production, although WKYMVM is more potent and was therefore used as control in our experiment (Karlsson et al., 2009). Our data show that SSL13 induced a modest oxidative burst compared with the control FPR2 specific peptide WKYMVM (Figure 4c), but both SSL13‐ and WKYMVM‐induced oxidative burst in human neutrophils could be blocked by FLIPr (Figure 4c). Furthermore, we tested whether SSL13 could induce neutrophil degranulation by measuring MPO activity in stimulated cell supernatant. MPO is one of the most abundant granule proteins in neutrophils and is efficiently released into the extracellular space during degranulation (Borregaard, 2010). Indeed, SSL13‐induced neutrophil degranulation (Figure 4d). Taken together, the functional outcomes of SSL13‐induced neutrophil activation include chemotaxis, ROS production, and neutrophil degranulation pointing towards a proinflammatory response of neutrophils to this staphylococcal protein.

To test whether SSL13 could act intracellular and is produced by *S*. *aureus* after uptake by human neutrophils, we generated a GFP promoter construct. Because SSL13 is part of an operon together with SSL12 and SSL14, the SSL12‐13‐14 promoter was cloned in front of GFP and transformed into *S*. *aureus* Newman. We did not observe expression of GFP under various standard culture conditions or after uptake of bacteria by phagocytes as seen with some other staphylococcal immune evasion proteins such as SPIN (de Jong et al., 2017) and PSMα (Surewaard et al., 2012; data not shown here).

### 2.5 SSL13 is not able to efficiently activate mouse neutrophils

**Figure 5:**
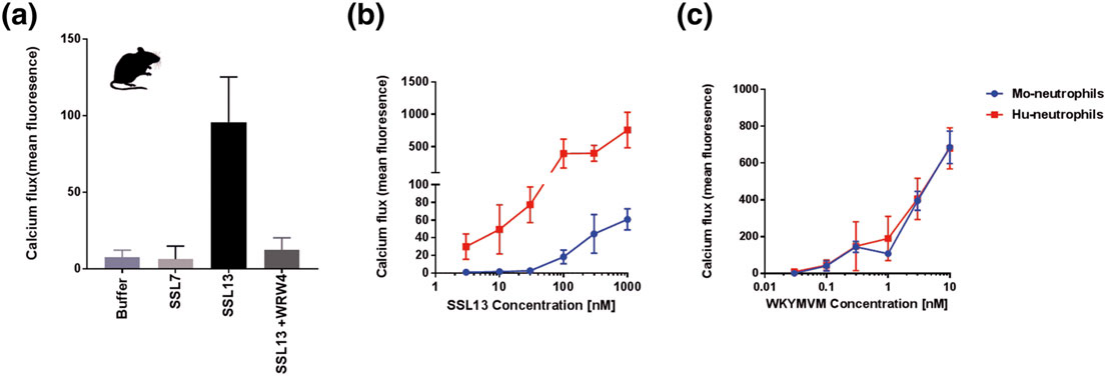
SSL13 is not able to efficiently activate mouse neutrophils. (a) SSL13 can induce activation of murine neutrophils, which can be inhibited by the mFPR2 antagonist WRW4. (b) SSL13‐induced calcium fluxes in murine neutrophils are low compared with human neutrophils. (c) WKYMVM‐induced calcium fluxes in murine neutrophils are similar to human neutrophils. Data are mean fluorescence ± SEM of three experiments

Many other staphylococcal immune evasion proteins show a high level of human specificity. In order to check the host‐dependent activation of SSL13, we tested binding and activation of neutrophils isolated from mice bone marrow. SSL13‐induced activation of murine neutrophils as shown by calcium mobilisation. Treatment of murine neutrophils with WRW4, a known inhibitor of murine FPR2 (Kretschmer et al., 2010), prevented SSL13‐induced calcium flux. This indicates that neutrophil activation by SSL13 happened in a murine FPR2‐dependent manner (Figure 5a), although much higher concentrations are needed as compared with human neutrophil activation (Figure 5b). In contrast, the specific FPR2 agonistic peptide WKYMVM showed similar activation ability to both human and murine neutrophils (Figure 5c). However, we were unable to detect any SSL13 binding to murine neutrophils (data not shown).

Because there was a minimal but specific activation of mouse neutrophils, we tested whether SSL13 can provoke a neutrophil influx after injection of SSL13 into the mouse‐abdominal cavity. We observed no increase in peritoneal neutrophil numbers at 4 hr after intra‐abdominal injection of 100‐μg SSL13 (data not shown). This indicates that SSL13 is highly adapted to specifically act on human neutrophils.

## 3 DISCUSSION

Previously, our group described a high‐throughput binding selection strategy using phage display, to identify S. aureus immune evasion molecules. In this strategy, only secreted proteins of a bacterial genome are expressed on the surface of a filamentous phage, which is well suited to identify and characterise immune evasion proteins (Fevre et al., 2014). Traditional phage selection strategies involve multiple rounds of selection and amplification and selecting single clones for sequencing and further analysis. Whole‐genome Illumina sequencing allows analysis of a phage library after only a single round of selection omitting library amplification that would undoubtedly lead to additional selection bias. Using this strategy, we identified 12 proteins involved in host–microbe interaction or immune evasion in a single round of selection indicating the enormous potential of this strategy. Furthermore, eight conserved hypothetical proteins identified need further characterisation and may also be involved in the host–microbe interaction. The identification of SSL13, a protein with previously unknown function, in this phage selection suggested an interaction between SSL13 and neutrophils. We show that SSL13 interacts specifically with FPR2, a member of the formyl peptide GPCR receptor family involved in recognition of MAMPs. The FPR2 is expressed by human neutrophils and SSL13 interaction leads to neutrophil activation and chemotaxis.

The SSLs are a family of 14 secreted proteins that were previously demonstrated to modulate immune evasion (Fraser & Proft, 2008; Jongerius et al., 2007; Koymans et al., 2016). Genetic analyses of 88 clinical S. aureus strains revealed that the genes encoding SSL12, SSL13, and SSL14 are conserved among all strains (McCarthy & Lindsay, 2013). We also confirm that SSL13 is produced in vivo as antibodies can be detected in human serum (Figure S4). Furthermore, in sharp contrast to the SSLs located on SPI‐2 that all have their own promoter, SSL12‐13‐14 share a single promotor. Our hypothesis is that SSL12‐13‐14 may be produced simultaneously by S. aureus under certain conditions and that their function could be linked. Unfortunately, we were unable to produce sufficient quantities of purified SSL12 and SSL14 to address this possibility. Just like the S. aureus bicomponent toxin PVL requires LukS‐PV and LukF‐PV to properly lyse neutrophils (Spaan, Henry et al., 2013), SSL12‐13‐14 might require the presence of all three proteins to elicit its maximum potential in immune modulation. Expression and secretion of most SSLs under standard culture conditions are very limited, and only low amounts of protein can be found in the cell culture supernatant. There is an up‐regulation and expression of some SSLs under different stress conditions (Torres et al., 2007). We also did not observe SSL13 expression, using a GFP promotor reporter construct, under standard bacterial cell culture or after uptake by neutrophils.

SSL13 is not the only secreted molecule from S. aureus that is able to activate neutrophils. PSMs, which are small peptides secreted by S. aureus, with a completely different structure compared with SSL13, are known to activate and attract both human and mouse neutrophils via FPR2 (Kretschmer et al., 2010; Surewaard et al., 2012; Weiss et al., 2017). In addition to this, micromolar concentrations of PSM have cell lytic activity, which is independent from FPR2. Serum can fully block PSMs functions in both the cell lysis and FPR2‐mediated neutrophil activation (Surewaard et al., 2012). However, SSL13 activity was not inhibited by serum and is not cytotoxic for neutrophils (Figure S5). In contrast to PSMs, SSL13 showed a high degree of human specificity and was not able to efficiently activate mouse neutrophils. Another difference is the regulation and expression of PSMs, which was shown to be induced after uptake by neutrophils (Surewaard et al., 2012). So we believe that this agonist is regulated differently from the PSMs, and thereby not a direct competitor for the same function via FPR2, but has its own yet unknown niche in the bacterial pathogenesis. Both PSMs and SSL13 require active synthesis, and maybe, sensing by the MAMP receptor FPR2 is an adaption of our immune system. For PSMs, production inside the neutrophil to lytic concentrations could contribute to escape of S. aureus to enable survival. SSL13 could have a yet unknown additional function, maybe in combination with the coexpressed SSL12 and SSL14.

FLIPr or its FLIPrL are located on the same immune evasion cluster 2 as SSL13, which are found in many, but not all, human S. aureus isolates (McCarthy & Lindsay, 2013). SSL13 is a neutrophil chemoattractant and activator that acts via FPR2, whereas FLIPr and FLIPrL bind and inhibit FPR2 signalling function (Prat et al., 2006; Prat et al., 2009). This may contribute to the ability of S. aureus to adjust a favourable balance between neutrophil activation and inhibition. Similar to other staphylococcal immune evasion proteins, many of the SSL proteins harbour several distinct functions. Therefore, it is not unlikely that SSL13 may have another unique function beyond activating FPR2 signalling. To conclude, SSL13 is a unique SSL member that does not belong to the immune evasion class but is a pathogen alarming molecule acting on FPR2.

## 4 EXPERIMENTAL PROCEDURES

### 4.1 Ethics statement

Informed consent was obtained from all subjects in accordance with the Declaration of Helsinki. Approval was obtained from the medical ethics committee of the University Medical Center Utrecht (METC‐protocol 07‐125/C approved March 01, 2010; Utrecht, The Netherlands). The use of animals was approved by the National Ethical Committee for Animal Experiments and performed according to the guidelines of the Central Animal Facility of the Utrecht University (permit AVD115002016565).

### 4.2 Reagents and antibodies

Monoclonal antibody (mAb) anti‐His tag (clone AD1.1.10, FITC‐labelled) was purchased from LS Biosciences, and anti‐CD62L (clone Dreg‐56, FITC‐labelled) and anti‐CD11b (clone ICRF44, APC‐labelled) were purchased from BD. The peptide MMK‐1 (H‐LESIFRSLLFRVM‐OH) was synthesised by Sigma, and WKYMVM was synthesised by Bachem AG (Switzerland). WRWWWW‐NH2 (WRW4) and Pertussis toxin (PTX) were purchased from Tocris. Formyl‐methionyl‐leucyl phenylalanine (fMLP), tumour necrosis factor α, and cytochalasin B were from Sigma‐Aldrich. Fluo‐3‐AM (acetoxymethyl ester) and Calcein‐AM were purchased from Thermo Fisher.

### 4.3 Cloning, expression, and purification of recombinant proteins

FLIPr, FLIPr‐like, and N‐terminal His‐tag labelled SSL13 (His‐SSL13) were cloned, expressed, and purified as described (de Jong et al., 2017; Prat et al., 2009). For SSL13, primers were designed without signal peptide according to the published sequence of the gene NWMN_1076 for cloning into modified N‐His6‐TEV‐(g)‐pRSET vector (de Jong et al., 2017). SSL13 was amplified from genomic DNA of S. aureus subsp. *aureus* strain Newman using the following primers: 5′‐CGGGATCCCAATTTCCTAATACACCTATC‐3′ and 5′‐ATATGCGGCCGCTTAGTTTGATTTTTCGAG‐3′. Restriction enzyme recognition sites are underlined. Recombinant protein was generated in *Escherichia coli* Rosetta Gami (DE3) plysS by induction with 1‐mM isopropyl β‐D‐1‐thiogalactopyranoside (Roche). His‐tagged protein was isolated under native purification conditions using a 5‐ml HiTrap chelating HP column (GE Healthcare) with an imidazole gradient (10–250 mM; Sigma‐Aldrich). The purified protein was analysed on a 12.5% SDS‐PAGE gel and showed one band corresponding to a mass of 26.8 kD (Figure S1). For direct fluorescent labelling, His‐SSL13 was mixed with 0.1‐mg ml^−1^ FITC (Sigma‐Aldrich) in 0.1‐M carbonate buffer (pH 9.5) for 1 hr at 4°C and subsequently separated from free FITC by overnight dialysis against PBS.

### 4.4 Cells

Human leukocytes were isolated from human heparinised blood as described (Fevre et al., 2014) and suspended in RPMI‐1640 supplemented with 20‐mM Hepes (Gibco) containing 0.05% HSA (Sanquin). HL60 cells were purchased from ATCC; HL60 cells stable transfected with the human‐FPR2 (HL60/FPR2) were kindly provided by F. Boulay (Laboratoire Biochimie et Biophysique des Systemes Integres, Grenoble, France). Cells were cultured in RPMI‐1640 supplemented with 10% fetal bovine serum, 100‐μg ml^−1^ streptomycin, and 100‐units ml^−1^ penicillin.

### 4.5 Phage library construction and phage production

A S. aureus secretome phage display library was created as described earlier (Fevre et al., 2014). Briefly, genomic DNA from S. aureus strain Newman was mechanically fragmented and fragments were cloned into the pDJ01 secretome phagemid vector (Fevre et al., 2014) and transformed into TG1 E. coli. Phages lacking an active pIII protein were produced overnight by coinfection with Hyperphage® helper phages (Progen) at a multiplicity of infection of 10. Phages were purified and concentrated using Polyethylene glycol (PEG) precipitation and resuspended in PBS to yield a final concentration of 2 × 10^11^ phages ml^−1^.

### 4.6 Phage selection on isolated human neutrophils

One millilitre of phage library was mixed with 1‐ml isolated human neutrophils (1 × 10^7^ cells in RPMI‐1640 + 0.05% HSA) and incubated on ice with gentle shaking for 30 min. Cells were washed twice by adding 50‐ml cold buffer and centrifugation. Phages were eluted using 500‐μl glycine 0.05 M, pH 2 for 5 min after which 62.5‐μl neutralisation buffer (2‐M Tris‐HCL pH 8.4) was added. Cells and cell debris were removed by centrifugation and phages were precipitated using 200 μl of 20% PEG/2.5‐M NaCl for 30 min at room temperature. Sample was centrifuged at 14,000 r.p.m. in an Eppendorf centrifuge for 10 min at 4°C, and supernatant was discarded. The pellet was suspended in 100‐μl iodide buffer (10‐mM Tris‐HCL, 1‐mM EDTA, 4‐M NaI, pH 8) to disrupt the phage coat proteins and release the DNA. DNA was precipitated by adding 250 μl of 100% ethanol and incubated for 30 min at room temperature. Sample was centrifuged at 14,000 r.p.m. in an Eppendorf centrifuge for 10 min at 4°C after which the supernatant was discarded and the pellet containing the single‐stranded phage DNA was washed with 70% ice cold ethanol and dried to the air. The nonselected phage library was taken as a control.

### 4.7 Phage library sequencing

Because the phage library was created using a pIII deficient helper phage, it consists of non‐infectious phage particles. Therefore, traditional phage selection with multiple rounds of selection and amplification is not possible and the library was analysed by genome sequencing using the Illumina MiSeq System. In order to add the MiSeq adapters to the isolated phage DNA, a polymerase chain reaction was performed on the precipitated DNA using Phusion® HF Polymerase (New England Biolabs), according to the manufacturer's recommendations. The primers were designed for compatibility with the Illumina MiSeq v2 sequencing kit (Table S1 for primer sequences). The polymerase chain reaction product was purified using gel purification on an Ultra‐pure 2% agarose gel, and the purified DNA was quantified on a Qubit 4 Fluorometer (Thermo Fischer Scientific). The purified sample was run on a 1% agarose gel to determine purity and determine mean fragment size.

Sequencing was performed by loading 3pM of the library onto a MiSeq v2 2x250bp sequencing kit and ran on an Illumina MiSeq System according to manufacturer's instructions. Sequence data were deposited in ENA under study accession number: PRJEB26168.

### 4.8 His‐SSL13 binding assay

To determine the binding of His‐SSL13 to human leukocytes, a mixture of isolated neutrophils and mononuclear cells at 5 × 10^6^ cells ml^**−1**^ was incubated with increasing concentrations of His‐SSL13 for 30 min at 4°C while gently shaking. Cells were washed and incubated with FITC‐labelled anti‐His‐tag mAb while shaking. Cells were washed and resuspended in buffer containing 1% paraformaldehyde (PFA). The fluorescence was measured on a FACSVerse flow cytometer, and the different leukocyte populations (neutrophils, monocytes, and lymphocytes) were identified on the basis of forward and sideward scatter parameters.

To determine the binding of His‐SSL13 to HL60 cells, 5 × 10^6^ cells ml^−1^ were incubated with FITC‐labelled SSL13 (SSL13‐FITC) for 30 min at 4°C while shaking. Cells were washed and resuspended in buffer with 1% PFA. The fluorescence was measured by flow cytometry, and cell populations were identified based on forward and sideward scatter parameters excluding debris and dead cells.

### 4.9 CD11b and CD62L expression on neutrophils

Neutrophils (5 × 10^6^ cells ml^−1^) were incubated with different concentrations SSL13 for 30 min at 37°C. Subsequently, the cells were put on ice and incubated with anti‐CD11b and anti‐CD62L mAb for 45 min on ice. Cells were washed and fixed with 1% PFA in buffer. Expression of CD11b and CD62L was measured on a flow cytometer and data expressed relative to the buffer treated cells.

### 4.10 Calcium flux in neutrophils and HL60 cells

Calcium flux with isolated human neutrophils and HL60 cells was performed in a flow cytometer as previously described (Prat et al., 2006). Briefly, cells at 5 × 10^6^ cells ml^−1^ were labelled with 0.5‐μM Fluo‐3‐AM ester, washed, and resuspended to a concentration of 1 × 10^6^ cells ml^−1^. To measure cells continuously and be able to add stimulus without interruption in the FACSVerse flow cytometer, the Eppendorf tube adapter was used without tube while sampling cells from a 96‐well plate on an elevated platform. Stimuli were added in a 1/10th sample volume after a 10‐s baseline recording and calcium flux monitored for 50 s post stimulation. Samples were analysed after gating neutrophils, thereby excluding cell debris and background noises. Calcium flux was expressed as difference between baseline fluorescence (mean of time point 3 till 8 s) and after addition of stimulus (mean of time point 20 till 60 s).

### 4.11 Chemotaxis

Neutrophil migration was measured in a 96‐multiwell transmembrane system (ChemoTX; Neuro Probe) using an 8‐μm pore size polycarbonate membrane (Bestebroer et al., 2009). Cells were labelled with 2‐μM calcein‐AM for 20 min and resuspended to a concentration of 2.5 × 10^6^ cells ml^−1^ in HBSS with 1% HSA. Wells were filled with 29 μl of chemoattractant, and the membrane holder was carefully assembled. Cells were preincubated with or without FLIPr, and 25 μl was placed as a droplet on the membrane. After incubation for 30 min at 37°C in a humidified 5% CO_2_ atmosphere, the membrane was washed extensively with PBS to wash away the nonmigrating cells, and the fluorescence was measured in a fluorescence plate reader (CLARIOstar; BMG LABTECH) using 483‐nm excitation and 530 emission filters. Percentage migration was calculated relative to wells containing the total fluorescence value of 25‐μl cells.

### 4.12 Myeloperoxidase (MPO) release

Neutrophils were treated for 10 min with cytochalasin‐B and tumour necrosis factor α with gently shacking and, without wash, subsequently incubated with buffer only, SSL13, or fMLP. Cells were centrifugated at 500×*g* for 10 min and supernatant collected for MPO activity measurement (van Kessel, van Strijp, & Verhoef, 1991). Therefore, 10‐μl sample was mixed with o‐dianisidine substrate and H_2_O_2_ in phosphate buffer at pH 6.0 and measured continuously for 30 min at 37°C in a plate reader (FLUOstar Omega) at 450 nm.

### 4.13 Neutrophil oxidative burst assay

Horseradish peroxidase and isoluminol were used as a sensitive measure of the human neutrophil oxidative burst as described (Dahlgren, Karlsson, & Bylund, 2007; Önnheim, Bylund, Boulay, Dahlgren, & Forsman, 2008). In white 96‐well microtiter plates, a 150‐μl reaction mixture of 6.25 × 10^4^ neutrophils per well in IMDM buffer with 0.1% HSA plus 50‐μM isoluminol and 4‐U ml^−1^ horseradish peroxidase was equilibrated for 5 min. Subsequently, concentrated stimulus was added to activate the NADPH oxidase and emitted light immediately recorded continuously for 15 min in a Luminometer (Berthold) at 37°C. Data are expressed as relative light units.

### 4.14 Mouse experiments

In the mouse peritonitis model, 100‐μg SSL13 in 0.5‐ml PBS was injected into the peritoneum of 6‐ to 8‐week‐old female CD‐1 mice. At 4 hr later, the mice were euthanised by cervical dislocation and abdominal cavities washed two times with 5 ml of RPMI medium containing 0.1% HSA and 5‐mM EDTA. In total, 8 to 9 ml of peritoneal fluid was recovered and centrifuged at 1,200 r.p.m. for 10 min to collect the exudate cells. Cell pellets were resuspended in 500‐μl buffer and counted with trypan blue in a TC20 automated cell counter (Bio‐Rad). Before immunostaining, cells were first preincubated with 100‐μg ml^−1^ normal goat IgG for 15 min. We stained the samples with APC‐conjugated antibody to mouse CD45 (leukocytes marker), PE‐conjugated antibody to mouse Gr1 (neutrophil marker), and FITC‐conjugated antibody to mouse F4/80 (macrophage marker). Samples were analysed on a flow cytometer.

Mouse neutrophils were isolated from bone marrow as described previously (Boxio, Bossenmeyer‐Pourié, Vanderesse, Dournon, & Nüsse, 2005). Briefly, a bone marrow cell suspension was collected by flushing the femurs and tibias with 10 ml of cold HBSS + 15‐mM EDTA + 30‐mM Hepes + 0.1% HSA. A two‐layer Percoll density gradient (2 ml each in PBS) composed of 81% and 62.5% was used to enrich neutrophils from the total leucocyte population. Interphase between 62.5% and 81% was collected. Cells were washed once with buffer and resuspended in RPMI‐1640 with 0.1% HSA.

Calcium fluxes in mouse neutrophils were determined as described above for human neutrophils with final concentrations of 10, 3, 1, 0.3, 0.1, and 0.03 nM of WKYMVM and 1,000, 300, 100, 30, 10, and 3 nM of SSL13.

Mouse neutrophil‐binding assays were conducted essentially as described for human neutrophils.

## Supporting information

Fig S1. Cloning, expression, and purification of SSL13.

Fig S2. SSL13 alters human neutrophils forward scatter. Peripheral blood leukocytes were incubated with buffer or 370 nM His‐SSL13 for 30 min at 37°C. Cell scatters were detected and analyzed by flow cytometry. The different cell populations were identified based on scatter parameters (A and B). SSL13 increases neutrophils forward scatter compared with untreated cells (C). Data are from one representative experiment.

Fig S3. SSL13 specifically interacts with human neutrophils. His‐SSL13 incubated with or without proteinase K for 30 min at 37°C. (A) SSL13 is degraded by proteinase K. (B) Degradation of SSL13 by proteinase K completely abolished the neutrophil binding. Neutrophil activation was measured by CD11b (C) and CD62L expression (D) Cell activation by SSL13 is inhibited by pretreatment with proteinase K. Data are mean fluorescence ± SEM of three experiments.

Fig S4. Detection of SSL13 serum antibodies. His‐SSL13 and His‐SCIN was coated to an ELISA plate overnight at 4°C. After incubation with a three‐fold dilution series of healthy human pooled serum, binding of human serum antibodies was detected using a goat‐anti‐human IgG‐HRP antibody. His‐SCIN, another secreted S. aureus protein, is a positive control. Data is from one representative experiment.

Fig. S5. SSL13 has no cytotoxic activity. Human neutrophils were incubated with PSM or SSL13 in presence or absence of serum. The membrane impermeable DNA stain DAPI was used to label dead cells. Phenol Soluble Modulins (PSM) are cytotoxic and this toxicity is inhibited by preincubation with serum. SSL13 is not cytotoxic as incubation with SSL13 does not lead to an increase in DAPI signal.

Table S1. Primers for genome sequencing
Table S2. Proteins with the highest read frequency after phage display selection
