# Supplementary material for: Staphylococcal superantigen‐like protein 13 activates neutrophils via formyl peptide receptor 2
Source: Cell Microbiol. 2018 Sep 17;20(11):e12941. doi: 10.1111/cmi.12941 (PMC6220968; doi:10.1111/cmi.12941)
Supplement: Supplementary file 1 — Fig S1. Cloning, expression, and purification of SSL13. [file CMI-20-na-s002.pdf]

**Table S1. Primers for genome sequencing**

|  | primers |
| --- | --- |
| pDJ01NextN701 | CAAGCAGAAGACGGCATACGAGATTCGCCTTAGTCTCGTGGGCTCGGAGATGTGTATAAGAGACAGCAGGACAATCCTGAACGCAGAAATCAAGAGG |
| pDJ01NextN702 | CAAGCAGAAGACGGCATACGAGATCTAGTACGGTCTCGTGGGCTCGGAGATGTGTATAAGAGACAGCAGGACAATCCTGAACGCAGAAATCAAGAGG |
| pDJ01NextN501 | AATGATACGGCGACCACCGAGATCTACACTAGATCGCTCGTCGGCAGCGTCAGATGTGTATAAGAGACAGCAAAATCACCGGAACCAGAGCCACCACCC |
| pDJ01NextN502 | AATGATACGGCGACCACCGAGATCTACACCTCTCTATTCGTCGGCAGCGTCAGATGTGTATAAGAGACAGCAAAATCACCGGAACCAGAGCCACCACCC |

**Table S2. Proteins with the highest read frequency after phage display selection**

| **Identified Protein** | **read freq.** | **# of clones** | **Protein function** |
| --- | --- | --- | --- |
| PTS mannitol transporter subunit IICB | 883 | 7 |  |
| Peroxidase Inhibitor | 196 | 4 | Inhibits human neutrophil myeloperoxidase |
| NWMN_0791 conserved hypothetical protein (HlyC/CorC family transporter) | 109 | 4 | Conserved hypothetical protein |
| Immunoglobulin G-binding protein Sbi | 101 | 2 | Inhibits opsonophagocytosis |
| Iron ABC transporter | 79 | 2 |  |
| Superantigen-like protein SSL4 | 72 | 7 | Binds sialated glycoproteins on myeloid cells |
| Acetyl-CoA acetyltransferase | 54 | 3 |  |
| Methicillin resistance protein FmtB | 50 | 3 |  |
| TIGR01440 family protein | 47 | 1 | Conserved hypothetical protein |
| Chemotaxis Inhibitory Protein | 41 | 2 | Inhibits C5aR and FPR1 mediated chemotaxis |
| Mannitol transporter protein | 39 | 1 |  |
| Meticillin resistance cassette | 39 | 4 |  |
| FPR2 inhibitory protein | 36 | 2 | Inhibits FPR2 on neutrophils and monocytes |
| Cell wall associated fibronectin-binding protein | 48 | 3 | Binds plasminogen and elastin |
| N-acetylmuramoyl-L-alanine amidase | 26 | 3 |  |
| Ribosomal RNA methyltransferase FmrO domain protein | 25 | 1 |  |
| CBS domain protein | 24 | 1 |  |
| Dihydroorotase | 19 | 1 |  |
| DUF4064 domain-containing protein | 19 | 1 |  |
| ATP-dependent Clp protease, ATP-binding subunit ClpC | 16 | 1 |  |
| Non-coding region between hypothetical proteins | 16 | 1 |  |
| NWMN_0280 conserved hypothetical protein similar to ORF061 of Bacteriophage ROSA | 15 | 1 | Conserved hypothetical protein |
| Aldehyde dehydrogenase family protein | 14 | 1 |  |
| Capsular polysaccharide biosynthesis protein CapJ | 14 | 1 |  |
| Conserved hypothetical protein (downstream LukED) | 14 | 1 | Conserved hypothetical protein |
| NWMN_2283 hypothetical protein DUF4889 superfamily(upstream conserved hypot 2282) | 14 | 1 |  |
| Fibronectin binding protein | 13 | 1 | Binds plasminogen and elastin |
| Intergenic region between BAF68719.1 en BAF68720.1 | 13 | 1 |  |
|  |  |  |  |
| Phage anti-repressor for bacteriophage phiNM1 | 13 | 1 |  |
|  |  |  |  |
| Branched-chain amino acid transporter II carrier protein | 12 | 1 |  |
| Conserved hypothetical protein similar to 5'-nucleotidase family protein multifunctional 2',3'-cyclic-nucleotide 2'-phosphodiesterase/5'-nucleotidase/3'-nucleotidase | 12 | 1 | Conserved hypothetical protein |
| Enterobactin ABC transporter permease | 12 | 1 |  |
| LLM class flavin-dependent oxidoreductase | 12 | 1 |  |
| Multidrug resistance transporter protein B | 12 | 1 |  |
| NWMN_2018 conserved hypothetical protein | 12 | 1 | Conserved hypothetical protein |
| Staphyloxanthin biosynthesis protein | 12 | 1 | Inhibits oxygen radicals |
| Bi-component leukocidin LukGH subunit H | 11 | 1 | Leukotoxin |
| Clumping factor A | 11 | 1 | Binds fibrinogen |
| Cysteine protease staphopain B | 11 | 1 | Inactivates neutrophil elastase and complement proteins |
| NWMN_0338 conserved hypothetical protein | 11 | 1 | Conserved hypothetical protein |
| Monovalent cation/H+ antiporter subunit D | 10 | 1 |  |
| Na+/alanine symporter family protein | 10 | 1 |  |
| NWMN_0344 conserved hypothetical protein (ABC-2 transporter permease) | 10 | 1 | Conserved hypothetical protein |
| Bi-component gamma-hemolysin HlgAB subunit A | 9 | 1 | Leukotoxin |
| Iron-regulated heme-iron binding protein IsdB | 9 | 1 |  |
| Lantibiotic leader peptide processing serine protease | 9 | 1 |  |
| Minor structural protein for bacteriophage phiNM3 | 9 | 1 |  |
| NWMN_0218 staphyloxanthin biosynthesis protein | 9 | 1 |  |
| NWMN_1584 conserved hypothetical protein | 9 | 1 | Conserved hypothetical protein |
| Peptidase M23B | 9 | 1 |  |
| Secretory antigen precursor SsaA homolog | 9 | 1 |  |
| Superantigen-like protein SSL13 | 9 | 1 | Activates FPR2 on neutrophils and monocytes |
| 16S rRNA methyltransferase | 8 | 1 |  |
| Manganese ABC transporter substrate-binding protein | 8 | 1 |  |
| MarR family transcriptional regulator | 8 | 1 |  |
| Multifunctional 2',3'-cyclic-nucleotide 2'-phosphodiesterase/5'-nucleotidase/3'-nucleotidase | 8 | 1 |  |
| rRNA-23S ribosomal RNA | 8 | 1 |  |
| Truncated transposase for IS1272 | 8 | 1 |  |
| Truncated triacylglycerol lipase precursor | 8 | 1 |  |

Table S2. **Proteins with the highest read frequency after phage display selection.** A *Staphylococcus aureus* phage display library was selected for binding against isolated human neutrophils. The selected library was analyzed by whole genome sequencing. Table shows the top hits after selection based on read frequency and grouped by number of different clones. Function of previously characterized immune evasion proteins and hypothetical proteins are listed.
