# Supplementary figures and images for: Staphylococcal superantigen‐like protein 13 activates neutrophils via formyl peptide receptor 2
Source: Cell Microbiol. 2018 Sep 17;20(11):e12941. doi: 10.1111/cmi.12941 (PMC6220968; doi:10.1111/cmi.12941)

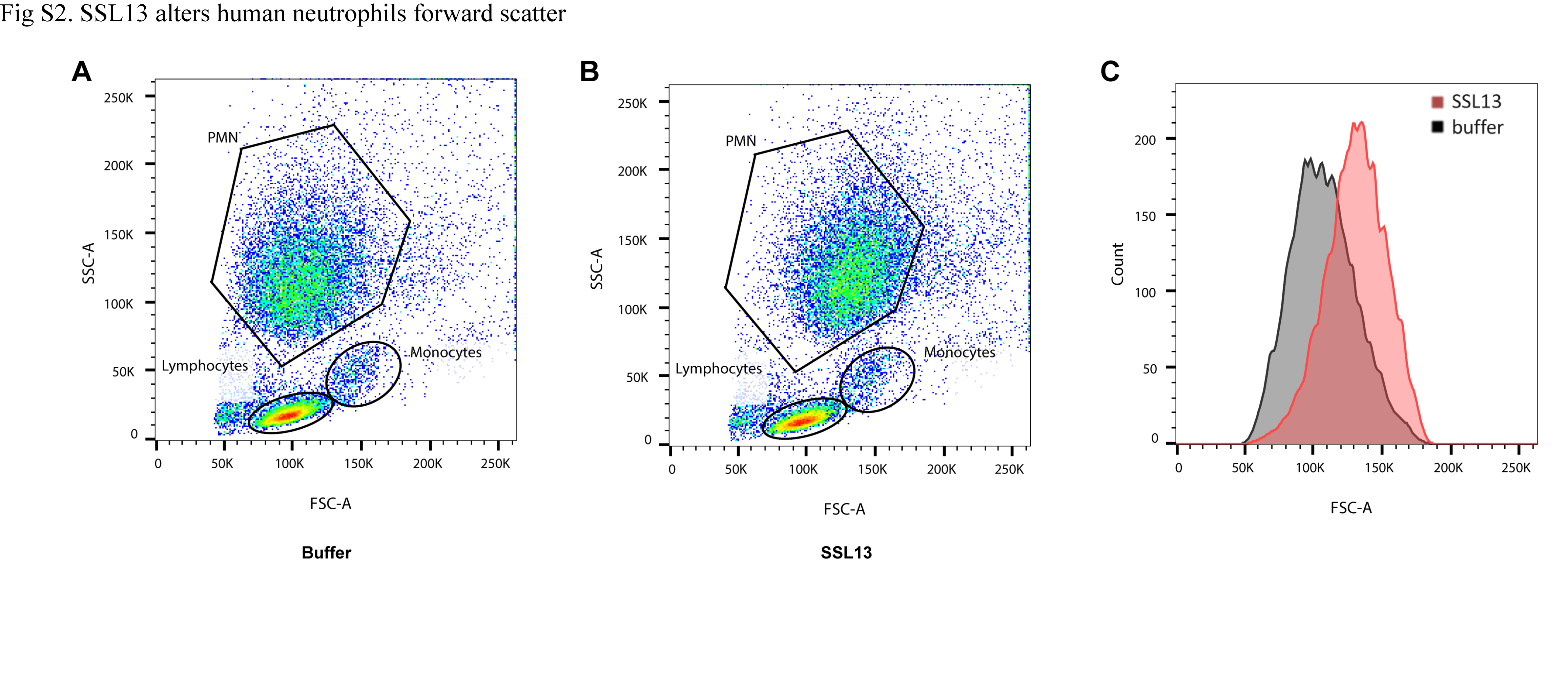

Supplement: Supplementary file 2 — Fig S2. SSL13 alters human neutrophils forward scatter. Peripheral blood leukocytes were incubated with buffer or 370 nM His‐SSL13 for 30 min at 37°C. Cell scatters were detected and analyzed by flow cytometry. The different cell populations were identified based on scatter parameters (A and B). SSL13 increases neutrophils forward scatter compared with untreated cells (C). Data are from one representative experiment. [file CMI-20-na-s003.tif]

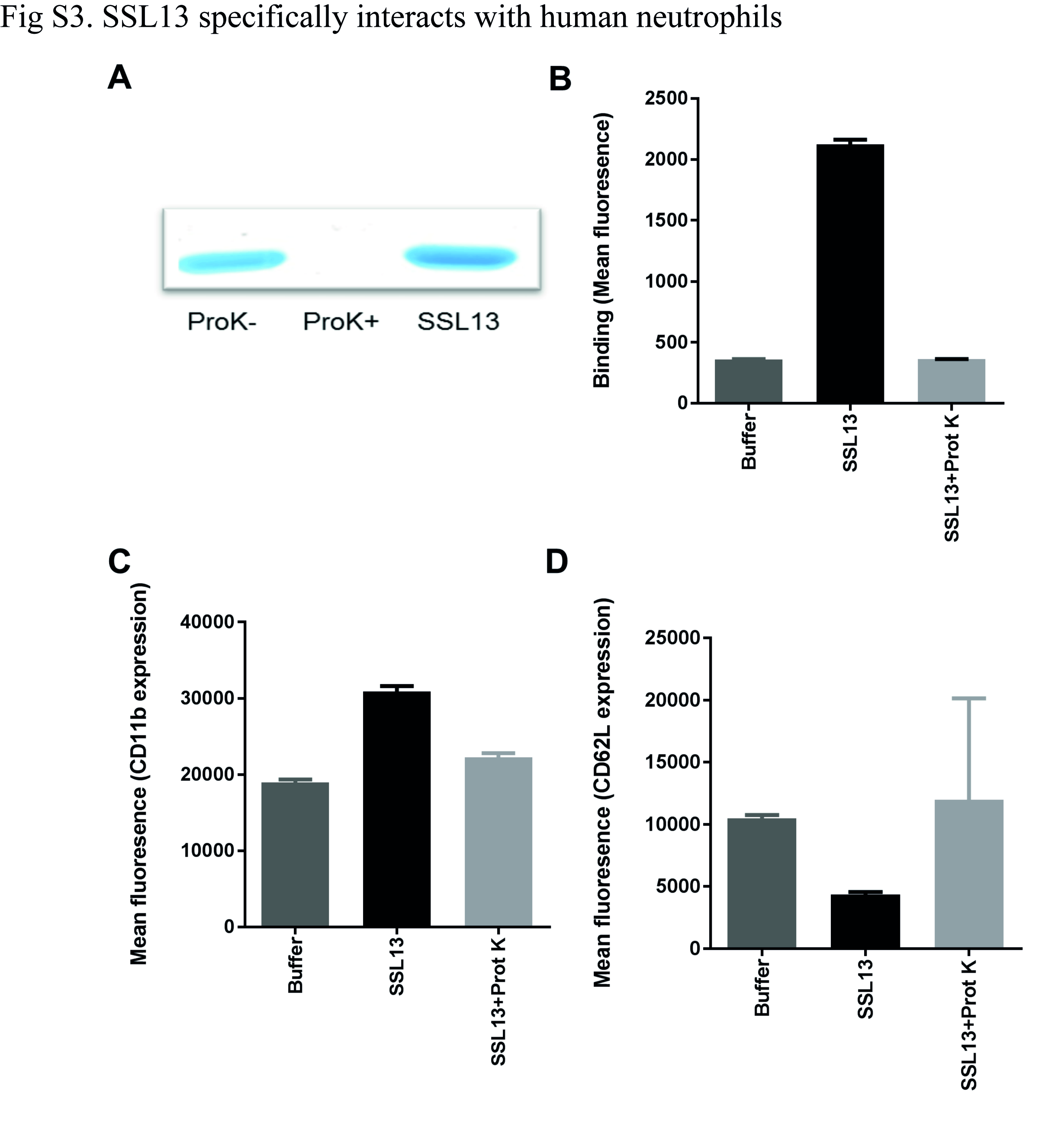

Supplement: Supplementary file 3 — Fig S3. SSL13 specifically interacts with human neutrophils. His‐SSL13 incubated with or without proteinase K for 30 min at 37°C. (A) SSL13 is degraded by proteinase K. (B) Degradation of SSL13 by proteinase K completely abolished the neutrophil binding. Neutrophil activation was measured by CD11b (C) and CD62L expression (D) Cell activation by SSL13 is inhibited by pretreatment with proteinase K. Data are mean fluorescence ± SEM of three experiments. [file CMI-20-na-s004.tif]

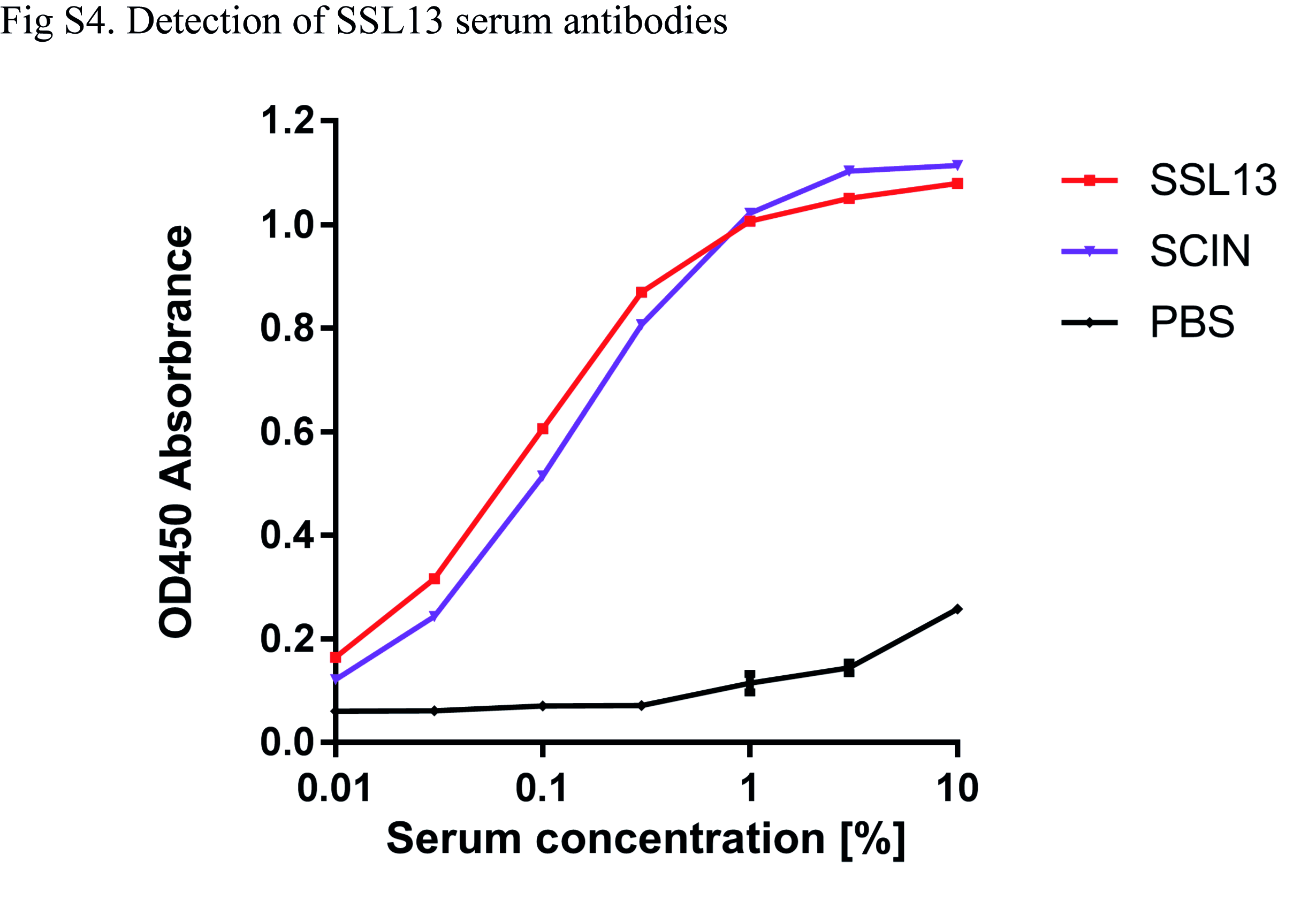

Supplement: Supplementary file 4 — Fig S4. Detection of SSL13 serum antibodies. His‐SSL13 and His‐SCIN was coated to an ELISA plate overnight at 4°C. After incubation with a three‐fold dilution series of healthy human pooled serum, binding of human serum antibodies was detected using a goat‐anti‐human IgG‐HRP antibody. His‐SCIN, another secreted S. aureus protein, is a positive control. Data is from one representative experiment. [file CMI-20-na-s005.tif]

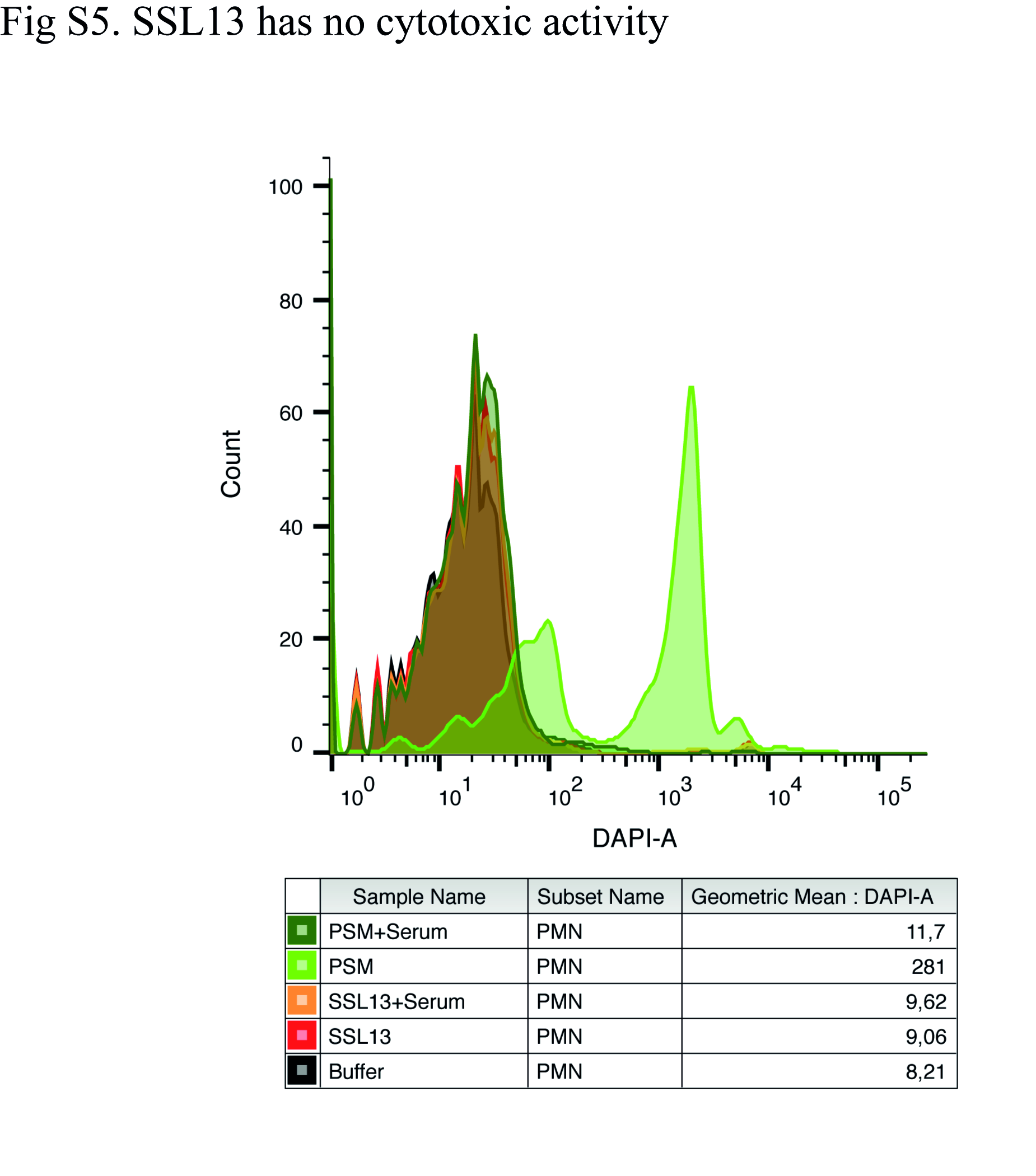

Supplement: Supplementary file 5 — Fig. S5. SSL13 has no cytotoxic activity. Human neutrophils were incubated with PSM or SSL13 in presence or absence of serum. The membrane impermeable DNA stain DAPI was used to label dead cells. Phenol Soluble Modulins (PSM) are cytotoxic and this toxicity is inhibited by preincubation with serum. SSL13 is not cytotoxic as incubation with SSL13 does not lead to an increase in DAPI signal. [file CMI-20-na-s006.tif]
