# Supplementary material for: Staphylococcal superantigen‐like protein 13 activates neutrophils via formyl peptide receptor 2
Source: Cell Microbiol. 2018 Sep 17;20(11):e12941. doi: 10.1111/cmi.12941 (PMC6220968; doi:10.1111/cmi.12941)
Supplement: Supplementary file 6 — Table S1. Primers for genome sequencing Table S2. Proteins with the highest read frequency after phage display selection [file CMI-20-na-s001.docx]

S1. Cloning, expression, and purification of SSL13

- His-SSL13 sequence

  MHHHHHHENLYFQGSQFPNTPINSSSEAKAYYINQNE
  TNVNELTKYYSQKYLTFSNSTLWQKDNGTIHATLLQF
  SWYSHIQVYGPESWGNINQLRNKSVDIFGIKDQETIDS
  FALSQETFTGGVTPAATSNDKHYKLNVTYKDKAETFT
  GGFPVYEGNKPVLTLKELDFRIRQTLIKSKKLYNNSY
  NKGQIKITGADNNYTIDLSKRLPSTDANRYVKKPQNA
  KIEVILEKSN

- Number of amino acids: 233
- Molecular weight: 26814.88
- Theoretical pI: 9.08
- Removed 1-23aa signal peptide
